# Impacts of COVID-19 on family violence in Thailand: prevalence and influencing factors

**DOI:** 10.1186/s12905-023-02440-x

**Published:** 2023-06-01

**Authors:** Wilai Napa, Nareemarn Neelapaichit, Ronachai Kongsakon, Somporn Chotivitayataragorn, Umaporn Udomsubpayakul

**Affiliations:** 1 Ramathibodi School of Nursing Faculty of Medicine Ramathibodi Hospital, Faculty of Medicine Ramathibodi Hospital, Mahidol University, Bangkok, Thailand; 2 Department of Psychiatry, Faculty of Medicine Ramathibodi Hospital, Mahidol University, Bangkok, Thailand; 3 Department of Nursing, Faculty of Medicine Ramathibodi Hospital, Mahidol University, Bangkok, Thailand; 4 Department of Clinical Epidemiology and Biostatistics, Faculty of Medicine Ramathibodi Hospital, Mahidol University, Bangkok, Thailand

**Keywords:** Family violence, Pandemic, COVID-19, Thailand

## Abstract

**Background:**

The COVID-19 pandemic drastically affected societies globally, prompting rising unemployment, insufficient household incomes, and stress and undermining women's and children's health within families. This study examined family violence and identified influencing factors during the COVID-19 pandemic in Thailand.

**Methods:**

A mixed-method design was used, entailing a questionnaire followed by focus group interviews. A cross-sectional survey was administered to investigate family violence among 1285 female respondents aged 15 years and above who were recruited through stratified sampling. The Cronbach alpha and and inter-raters Kappa coefficient values for the questionnaire were 0.67 and 1.00, respectively. In addition, a descriptive qualitative instrument was employed to analyze the data sets from four focus group interviews held with 32 staff members from agencies that deal with family violence. The researchers jointly developed the focus group questions, which focused on the impacts of the COVID-19 pandemic on family violence. They independently analyzed data using content analysis.

**Results:**

The majority of the study participants were aged above 45 years (>50%), married (61.1%), lived in single-family settings (52.5%), had lost their jobs (64.4%), and had economic constraints that were moderate (37.8%) to severe (40.6%). The prevalence of family violence, which was primarily physical, was 42.2%. Family income, stress, and substance abuse were the main factors associated with family violence. These findings were correlated with those from the qualitative interviews.

**Conclusions:**

The COVID-19 pandemic had indirect impacts through family violence. Women were subjected to family violence behaviors, which were associated with household income, economic status, stress, and substance abuse. These behaviors included psychological and physical violence, as well as sexual abuse. Future interventions should focus on financial support and stress reduction.

**Supplementary Information:**

The online version contains supplementary material available at 10.1186/s12905-023-02440-x.

## Background

The World Health Organization (WHO) declared the novel coronavirus 2019 (2019-nCoV) pandemic as a public health emergency of international concern (PHEIC) following a meeting held to consider the situation on January 30, 2020 at the WHO headquarters in Geneva, Switzerland [1]. Because of mutations of the coronavirus, COVID-19 outbreaks have occurred in multiple waves [2]. Following the onset of pandemic, infections dramatically increased across the world, including in the United States, India, Brazil, Russia, the United Kingdom, France, Spain, Italy, Turkey, and Germany. The number of deaths, particularly among the elderly, also increased [3]. The number of confirmed infected patients in Thailand has continuously increased, commencing from 2020 [4].

During the COVID-19 pandemic, there was gradual increase in globally. The United Nations (UN) reported a global increase in violence against women and individuals within the families. For example, France reported a 30% increase in domestic violence, and in Argentina, there was 25% increase following the lockdown imposed by the government in March 2020 [5]. Multiple reports from China indicated that there has been an increase in family violence prompted by increased family conflict, economic distress, and inadequate social support for victims during the COVID-19 pandemic [6]. The pandemic also impacted parent–child interactions. Studies have shown that individuals who lose their jobs and income sources experience psychological distress, which is associated with adverse interactions which their children [7]. In addition, low-income and lower-middle-class individuals and those of color experienced higher level of mental and financial hardships as a results of the pandemic, which adversely affected their relationship with their children [8]. According to the Center for Disease Control and Prevention in Thailand, protocol regulations, including city lockdowns and layoffs, affected families and consequently psychological distress and their financial status, which were factors linked to increase family violence [9, 10].

Prior to the COVID-19 pandemic, a survey conducted in Thailand in 2018 showed that family violence occurred in approximately one out of three families, with income and substance abuse accounting for where 48.1% of domestic violence [11]. In addition, family violence was found to be more prevalence with in families belonging to hill tribes and among women and children living with family members who consumed alcohol [12]. Another study found that 15% of respondents experienced psychological, physical, or sexual violence and that 1 in 6 Thai women have faced intimate partner violence [13]. However, the impact of the COVID-19 pandemic on family violence in terms of its prevalence and related factors remains unclear. Therefore, this study aimed to explore family violence occurrence and the factors linked to women's and children's health that influence family violence.

## Methods

A mixed-method to explore the prevalence of family violence and influencing factors during the COVID-19 pandemic in Thailand. First a quantitative survey was perform to determine the prevalence and impact of family violence on women’s health. This was followed by qualitative focus group interviews conducted with staff working in agencies that deal with family violence.

### Quantitative component

 This study explored the prevalence of family violence and examined the impacts of the COVID-19 pandemic on the health of women living in a family setting. A cross-sectional interview-based survey was conducted to obtain on domestic (family) violence at the national level in provinces across central, northern, northeastern, and southern Thailand, including the capital city of Bangkok. The required sample size was calculated using Wayne'sformula [14] to guarantee a 95% confidence interval (CI) for detecting the prevalence of domestic violence in Thailand [11]. The target sample size that was initially calculated was 1065 households. However, the actual sample size in the study was approximately 20% higher to account for invalid questionnaires. Therefore, the total sample size was set at 1285 households. In addition, the number of families recruited in each province for the sample was calculated in proportion to its population provinces.

A stratified four-stage sampling method was used to select the target provinces. The first stage entailed random sampling of two provinces in each of the four regions along with Bangkok (amounting to a total of nine provinces). In the second stage of sampling, two districts, one urban and one rural, were randomly selected in each province. In the third stage, one sub-district in each district was randomly selected. In the final stage, households were randomly chosen from each sub-district or community. A woman in each family was deemed eligible to participate if they were aged 15 years and above and normally lived in the household with other family members. Participants were interviewed face to face with no family members present using a structured questionnaire.

Extensive inputs were sought from experts, who included a psychiatrist, a medical epidemiologist, nurses, and social workers, to develop a structured questionnaire focusing on family violence. Subsequently, others six experts relevant to family violence reviewed the instrument and suggested revisions to the questionnaire. The index of item objective congruence (IOC) of the resulting instrument was 0.5–1.00. A pilot study was performed with 154 women to determine the instrument’s reliability. The Cronbach’s alpha coefficient was 0.67, thus, confirming the instrument’s reliability and validity. The Kappa coefficient for interrater reliability for 15 interviewers was 1.00. The researchers analyzed the data and used descriptive statistics to determine the prevalence of domestic violence, and a logistic regression was performed to identify factors associated with domestic violence. The researchers conducted the survey using questionnaires to assess family violence during two phases of the COVID-19 pandemic: at baseline (January to May 2020) and at six-month follow-up (July to December 2020).

### Qualitative component

The qualitative component comprised focus group interviews to explore the perspectives of individuals working at agencies that deal with family violence regarding the nature and cause of family violence that occurred during the COVID-19 pandemic. Their suggestions on policy input were also elicit. Thirty-two individuals from agencies and organizations working with families experiencing violence, including village leaders, village volunteers, social development and human security volunteers, social development and human security officers, social workers, lawyers, nurses, police officers, and attorneysparticipated. All of these participants had been working in one of the four study provinces for six months or more prior to participating in the focus group interviews. Each focus group for each province comprised eight to ten persons. Before participating in the focus group, the participants signed informed consent forms in which they agreed to be tape-recorded.

The semi-structured questions for the focus groups were developed in line with the agreed objectives of the study using open-ended questions. An interviewer experienced in conducting qualitative research facilitated the focus group interviews, which lasted between 60 and 90 min. The recordings of the interviews were transcribed verbatim, and all transcripts were checked before being analyzed. Additionally, one individual took notes during the interviews, which were used to recheck the transcripts. Content analysis was performed on the focus group interview [15]. The first stage of data analysis comprised reading and rereading all the transcripts to make sense of the data. Open coding was initiated by highlighting, in different colours, words or phrases used during each conversation and responses linked to the research objectives, such as those relating to the impacts of the COVID-19 pandemic or the reasons for family violence. Next, inductive content analysis of the above text was performed. The text included in the open coding was then group into themes to explain how the COVID-19 pandemic affected families indirectly through family violence. At this point, the researchers also examined the relationships among categories to ensure that each of them was independent.

## Results

An analysis of the demographic data obtained for 1285 women who participated in the study revealed that 49.8% of the participants lived in cities and 50.2% lived in rural areas. Moreover, 52.5% lived in a single-family setting and 39.0% in an extended-family setting with the number of family members ranging from 2–20 persons (Mean ± SD; 4.1 + 1.9). More than 50% of the respondents were aged > 45 years, and 61.6% of them were married. A total of 53.5% of the participants had completed primary school (grade 6), 23.3% were housekeepers, and 22.3% were employees. The pandemic monthly incomes of 68.8% of the participants were below 10,000 Baht (approximately USD 300). Additionally, 7.9% of them stated that that their family incomes were usually insufficient to meet their daily expenses. During the pandemic, most participants (87.1%) had a monthly household income of less than 10,000 Baht, and 30.6% reported that their family incomes were generally insufficient to met their daily expenses. More than half (65.6%) of the participants stated that their family members smoked, consumed alcohol, or engaged in substance abuse (see Table 1).

**Table 1:** Characteristics of participants (*n* = 1285)

Characteristics	Number (%)
Residence	
Urban	640 (49.8)
Rural	645 (50.2)
Age (in years)	
15 – 29	45 (3.5)
30 – 44	196 (15.3)
45 – 59	617 (48.0)
60 – 69	321 (25.0)
70 – 79	95 (7.4)
80^+^	11 (0.9)
Mean ± SD	54.0 ± 11.7
Range	(15–86)
Marital status	
Single	118 (9.2)
Married	818 (63.7)
Separated	19 (1.5)
Divorced/widowed	330 (25.7)
Education	
No education	82 (6.4)
Primary education	687 (53.5)
Secondary education	160 (12.5)
Higher education	356 (31.3)
Occupation	
Laborer	287 (22.3)
Business owner	263 (20.5)
Agriculturist	347 (27.0)
Government officer	23 (1.8)
Company employee	20 (1.6)
Student	7 (0.5)
Housewife	299 (23.3)
No occupation	39 (3.0)
Pre-pandemic household income (in Baht)	
<5000	182 (14.2)
5000–10000	329 (25.6)
10001–20000	373 (29.0)
20001–30000	235 (18.3)
30001–30000	74 (5.8)
40001–50000	35 (2.7)
>50,000	30 (2.3)
Does not know	27 (2.1)
Adequate household income for expenses before the pandemic	
Adequate income with savings	237 (18.4)
Adequate income but no savings	648 (50.4)
Indigent	298 (23.2)
Inadequate	102 (7.9)
Household income (in Baht) during the pandemic	
<5000	423 (32.9)
5000–10000	425 (33.1)
10001–20000	271 (21.1)
20001–30000	76 (5.9)
30001–30000	34 (2.6)
40001–50000	16 (1.2)
>50,000	12 (0.9)
Does not know	28 (2.2)
Adequate household income for expenses during the pandemic	
Adequate income with savings	78 (6.1)
Adequate income but no savings	374 (29.1)
Indigent	440 (34.2)
Inadequate	393 (30.6)
Number of family members	
Mean ± SD	4.1 ± 1.9
Range	(2–20)
Type of family	
Nuclear	674 (52.5)
Extended	501 (39.0)
Single-parent	59 (4.6)
Skipped-generation	51 (4.0)
Smoking/alcohol consumption in the family	
Smoking	533 (41.5)
Alcohol consumption	577 (44.9)
Substance abuse in the family	
Kratom (Mitragynine)	4 (0.31)
Amphetamine	8 (0.62)
Not specified	5 (0.38)
Inhalants	1 (0.07)

The demographic data obtained during focus group interviews with 32 staff members from agencies that deal with family violence indicated age range of 30–70 years. Of these respondents, 56.3% were married, 50.0% had a bachelor's degree,and 56.4% were government officers. They provided their perspective on family violence involving women and children aged below 15 years, which, included physical as well as sexual abuse, resulting in physical and psychological distress. The following quotations have been extracted from the interviews: 

During the COVID-19 pandemic, they (families) faced living constraints, so they argued with each other. Often, this ended up with occurrences of physical assault. However, they didn’t take it any further or go to the police station.

Because the schools weren’t operating to teach students on-site while the country was in lockdown, the students attended classes online in their homes instead. As a result, they were sexually abused by parents who were using drugs or watching pornography or X-rated film.

### The impacts of the COVID-19 pandemic

Of the survey respondents 64.6% stated that the pandemic had led to their unemployment because they were laid off by their employers, while 21.9% stated that their businesses ceased operations. The economic impacts on families were severe (40.6%) to moderate (37.8%). A rating scale was used to determine the level of family stress and to evaluate whether families felt that they were living under pressure. The families’ responses revealed that they felt moderate to severe levels of stress (median=5), and they solved problems by talking to each other to relieve stress (Table 2).

**Table 2:** Impacts of the COVID-19 pandemic (*n* = 1285)

Impact	n (%)
**Impact on household employment**	
- Loss of job/working hours	830 (64.6)
- Unemployed (laid-off) / business closed down	281 (21.9)
**Economic impact on the household**	
- No impact to mild impact	278 (21.6)
- Moderate impact	486 (37.8)
- Severe impact	521 (40.6)
**Family stress score (0-10)**	
- Median (P_25_ – P_75_)	5 (3 – 7)
**The family had discussions/talked about the problem**	
- Did not have any discussions or there were discussions but conflicts occurred	236 (18.4)
- Had discussions/talked to solve problems	1049 (81.6)

The 32 staff members observed that during the COVID-19 pandemic, domestic violence most often occurred within families in which a family member lacked earning opportunities, leading to high stress levels and the use of drugs and heavy drinking. Consequently, they fought with family members, particularly female ones. In addition, some families having children who attended classes online at home were subjected to sexual abuse by their parents. The following excerpts from the interviews with participating staff members highlight these issues:

The COVID-19 pandemic affected families that had low incomes and members who were employees who lost their incomes, resulting in family violence. If they had debt and insufficient money to meet their expenditure, the stress and family problems would pile up.

Before the COVID-19 pandemic, the family members loved each other and didn’t fight with each other. However, they became violent and drank too much alcohol after the onset of pandemic.

Child abuse occurred because first, the children didn’t go to school (because school was closed) and second because their parents used drugs and were unemployed. Then parents got more stressed and, maybe, they watched pornography. This led to family violence and especially sexual abuse of both wives and children.

**Fig. 1:**
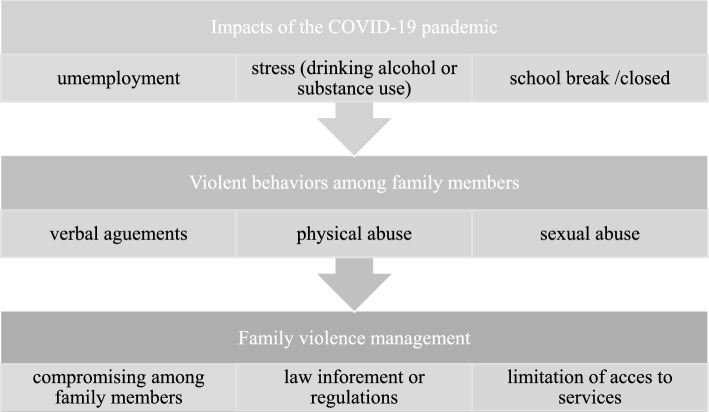
The COVID-19 pandemic situation and family violence

The descriptions of the agency staffs reflect the impacts of the pandemic, including unemployment and stress, which resulted in increased alcohol consumption and/or substance use. Additionally, the school lockdown was associated with an increased incidence of sexual abuse of children within households. Family violence increased and entailed physical and sexual abuse. Lastly, the staff reported that whereas verbal compromise and legal interventions served to relieve the consequences of the increases in family violence, the measures implemented to prevent the spread of COVID-19 limited the space available for sheltering or quarantining victims (Fig. 1).

### The prevalence of family violence

A total of 542 of 1285 households (42.2%) reported that they had experienced domestic violence during the pandemic. The most common type of violence reported was psychological abuse (41.2%), followed by physical abuse (4.3%), and, sexual abuse (2.3%), with several respondents reporting having suffered more than one type of violence (Table 3). The most common types of psychological violence were insultsor humiliation (86%), being ignored (33.8%), and threatening behaviors (15%). Physical violence reported by the respondents were being slapped, beaten, kicked, or trampled (60%) and being pushed, pulled, or thrown (40%). Of the respondents, 76.7% reported being forced to have sex (Table 4). In a second survey, conducted six months after the first survey, 406 of 1241 households (32.7%) stated that they had experienced domestic violence. Of the respondents, 32.0% experienced psychological abuse, 2.3% experienced physical abuse, and 1.0% experienced sexual abuse with respondents reporting more than one type of violence (Table 3).

**Table 3:** Prevalence and types of violenc

	(*n* = 1285)	6-month later (*n* = 1241)
	n (%)	n (%)
Experienced violence		
Never	743 (57.8)	835 (67.3)
Ever	542 (42.2)	406 (32.7)
Types of violence		
Psychological	530 (41.2)	397 (32.0)
Physical	55 (4.3)	29 (2.3)
Sexual	30 (2.3)	12 (1.0)

**Table 4:** Prevalence of violence by type of violence at baseline and after six-months

	Baseline	After 6-months
	n (%)	n (%)
Psychological violence	530 (100%)	397 (100%)
Insults, belittling, irritability, humiliation	456 (86.0)	334 (87.7)
Scaring tactics	17 (3.2)	12 (4.1)
Ignoring	179 (33.8)	95 (29.8)
Threatening behaviors	81 (15.3)	80 (25.0)
Brake a word	10 (1.9)	13 (4.4)
Infidelity	16 (3.0)	4 (1.4)
Dominating/controlling behavior	6 (1.1)	3 (1.0)
**Physical violence**	**55 (100%)**	**30 (100%)**
Pushed, pulled, thrown	22 (40.0)	13 (43.3)
Slapped, beaten, kicked, trampled	33 (60.0)	11 (50.0)
Threatened with weapons or their actual use	5 (9.1)	1 (3.3)
Others (undisclosed)	5 (9.1)	1 (3.3)
**Sexual violence**	**30 (100%)**	**12 (100%)**
Sexual harassment	2 (6.7)	7 (58.3)
Molestation/ obscene behavior	1 (3.3)	1 (8.3)
Forced to have sex	23 (76.7)	3 (25.0)
Others (undisclosed)	4 (13.3)	1 (8.3)

### Factors associated with family violence

The factors related to domestic violence during the pandemic included the family stress and smoking or alcohol consumption in the family after adjusting covariate variables such as income status, type of family structure, the economic impact on households, and substance abuse within the family (amphetamines, Kratom, inhalants, and unspecified substances) (Table 5). The factors associated with family violence included a high family stress scores (6–10), with OR of 1.987 (95% CI: 1.532–2.578); having a smoker in the family (OR = 1.456; (95% CI: 1.014–2.089), alcohol consumption within the family (OR =2.185; 95% CI: 1.574–3.034), and smoking and alcohol consumption within the family (OR= 1.669; 95% CI: 1.253–2.221).

**Table 5:** Factors associated with family violence

	Prevalence	Unadjusted OR (95% CI)	*Adjusted* OR (95% CI)
Income status during the pandemic			
Adequate income with and savings	30.8	1	-
Adequate income but no savings	37.4	1.346 (0.797–2.274)	-
Indigent	41.4	1.587 (0.947–2.661)	-
Inadequate	49.9	2.239 (1.331–3.765)	-
Type of family			
Nuclear	41.8	1	-
Extended	40.7	0.955 (0.755–1.208)	-
Single-parent	50.8	1.438 (0.844–2.450)	-
Skipped-generation	51.0	1.446 (0.818–2.556)	-
Economic impact on household			
No impact to mild impact	34.5	1	-
Moderate impact	41.6	1.348 (0.993–1.831)	-
Severe impact	46.8	1.670 (1.236–2.257)	-
Family stress score			
Score 0–5	33.9	1	1
Score 6–10	52.7	2.177 (1.737–2.730)	1.987 (1.532–2.578)
Smoking/alcohol consumption in the family			
None	34.2	1	1
Smoking	43.3	1.470 (1.039–2.079)	1.456 (1.014–2.089)
Alcohol consumption	51.8	2.072(1.507–2.850)	2.185 (1.574–3.034)
Smoking and alcohol consumption	47.6	1.752 (1.331–2.306)	1.669 (1.253–2.221)
Substance abuse in the family (amphetamine, Kratom, inhalants, not specified)			
No	41.8	1	-
Yes	66.7	2.781 (1.037–7.457)	-

The staff members provided supplementary information and their perspectives offered a deeper understanding of why family violence occurred during the COVID-19 pandemic, as illustrated in the following quotation from a focus group interview:

The causes of family violence before and during the COVID-19 pandemic were not different. This violence takes place when a family member drinks, uses drugs or even has an affair. Not having any money, or having insufficient money, is the main family problem that leads to physical abuse.

## Discussion

This study explored the prevalence of family violence inflicted on women and children in Thailand during the COVID-19 pandemic. The research was carried out using structured questionnaires and focus groups conducted among women who lived with their families. The data, which were collected from July 2020 to January 2021, covered the two waves of the pandemic and were used to compared the prevalence of family violence to determine whether there were any differences during these waves. While collecting data during the second wave of the pandemic, the perspectives and insights of staff members of agencies dealing with domestic violence were included to provide supplementary information and a greater depth of understanding of the pandemic’s impacts and the causes of family violence. Nine provinces across Thailand, namely Bangkok, Chonburi, Ratburi, Chiang Mai, Phitsanulok, Ubon Ratchathani, Udon Thani, Surathani, and Trang were selected for this study. The following sections focus on the prevalence of and factors associated with family violence during the pandemic.

### The prevalence of family violence affecting women and other family members during the pandemic

The prevalence of family violence in Thailand increased from 34.6% in 2017 [11] to 42.2% in 2021 during the pandemic, which correlates with the increased incidence of domestic violence reported in United Kingdom, Peru, Argentina, Bangladesh, Spain, and India [9]. Additionally, a study from England showed that 53.1% of the participants experiencing violent acts reported that the violent acts increased in frequency, number, and severity during the COVID-19 pandemic [16].

A survey conducted during period when the Thai government implemented regulations mandating social distancing and a lock down in the country found that the overall rate of unemployment increased from 1.0% in 2019 to 1.9% in 2021 and mainly affected those employed within the tourism, hospitality, and entertainment sectors [17]. Consequently, during the height of pandemic, companies closed down or else they downsized and laid off employees or reduced their working days or salaries. As a result, families were under economic constraints, particularly those whose household incomes decreased from 20,000 baht per month (US$600) to 10,000 baht per month (US$300), and 86.5% experienced unemployment. Therefore, these families had insufficient money to meet their daily living requirements and experienced stress which is likely to have increased the incidence of family violence and engagement in risky behaviors, including substance use and involvement in robberies, or other criminal activities.

A survey found that during a period of pandemic from January 2021 to January 2022, the prevalence of family violence decreased from 42.2% to 32.7%, and the unemployment percentage within families decreased from 86.5% to 76.6%. In addition, the government provided subsidies to lower-income families to help them to meet the cost of living. Governmental support included short-term compensation for insured persons in social security (employed and unemployed persons covered by social security), increasing national welfare, and short term compensation for lower-income families. Additionally, the number of people embarking on new careers, such as food delivery and selling food online, increased by 5%. Although macroeconomic factors, such as gross domestic product, gross national income, and inflation rates remain uncertain household incomes were subsidized by the government package [18]. Thus, that the finding of a decrease in family violence could be related to decreased economic and psychological stress.

Besides providing information about women’s health during the pandemic, the staff interviews revealed that the incidence of abuse of children under age of 15 years increased significantly. The Ministry of Education required schools to prepare for and swich to teaching classes online instead of on-site because of the regulations associated with the national lockdown. Consequently, some children attended classes online in their homes, and, were at risk for sexually or physically abused by their parents. These findings are consistent with the report of the International Labour Organization that highly-stressed parents subjected their children to verbal abuse and corporal punishment nationwide during the pandemic [19]. The staff members also noted that an increase in expressed emotions, such as fighting or arguing, and higher levels of stress resulted in the increased frequency and severity of violence within families already experiencing domestic violence.

### The factors associated with family violence during the pandemic

The finding of this study indicated that family stress and alcohol consumption were key triggers for family violence during the pandemic. The survey results and focus group interviews showed that families with financial constraints were likely to experience increased family violence. The finding that Thai families faced increased stress and engaged in more verbal aggression during the nationwide lockdown caused by the pandemic [19] endorse those of a British study, which reported that significant psychological problems were experienced during the pandemic [20]. Therefore, during the pandemic, financial constraints were likely to cause increased family stress and depression, thus compounding verbal aggression and physical abuse.

The survey results and focus group interviews in the present study that alcohol consumption or substance abuse tend to induce family violence. Previous studies have shown that the risk of family violence is 3.4 times higher in a family with a member whose of alcohol consumption is high than in families in which alcohol consumption is not high [20]. Moreover, level of verbal and physical abuse are higher in families whose members consume alcohol [19]. Whereas, alcohol consumption and drug dependency may not be directly associated with domestic violence, they are potentially implicated in cases involving severe domestic violence [21, 22]. Alcohol has been directly linked to cognitive dysfunction, particularly poor judgment and impulse control, which are factors contributing to a violent act [23]. The bottom line is that alcohol consumption and substance abuse may indirectly influence family violence.

Recommendations for attenuating increase domestic violence as a consequence of the COVID-19 pandemic center on three main factors: financial/economic support, stress management and reduction, and access to services. First, the provision of financial support to those with no incomes or inadequate incomes should be prioritized. Such support includes providing land to grow crops, low-interest loans, and subsidies to help meet living expenses. Next, to reduce stress within families, programs, and interventions should be developed that focus on teaching vulnerable people how to use adaptive coping skills to manage stress, instead of increasing their alcohol consumption. In addition, families in which there is a risk of violence occurring should be able to access services easily, and measures should be implemented to promote early detection along with interventions, and access to support services. Additionally, the government should allocate sufficient space for promptly quarantining the victims of domestic violence in a safe location.

## Conclusion

The COVID-19 pandemic significantly impacted Thai society, leading not only to direct consequences from the disease itself but also to significant impacts on the business sector. The prevalence of family violence in Thailand was found to have increased from 34.6% in 2017 to 42.2% in 2021. Many businesses in the tourism and manufacturing sectors laid-off employees or closed down because of the mandates instituted to prevent the spread of disease. Consequently, unemployment increased, with corresponding decrease in employees’ earned incomes, which caused financial constraints. Therefore, employees who had insufficient incomes were more likely to have increased stress and to engage in alcohol consumption and substance abuse, which are likely to increase violence inflicted on women and children within the families.

### Limitations of the study

This study had some limitations, including an absence of the perspectives of the family experiencing violence. Some dimensions, such as service user were also not covered. Nevertheless, its provides new information on the effects of the COVID-19 pandemic on families in Thailand, although its findings for this population should be interpreted with caution.

## Supplementary Information

**Additional file 1.**

**Additional file 2.**

## Data Availability

Data used in this study are available from the corresponding author on reasonable request.
