# Correction: Impacts of COVID-19 on family violence in Thailand: prevalence and influencing factors

**DOI:** 10.1186/s12905-025-04141-z

**Published:** 2025-11-27

**Authors:** Wilai Napa, Nareemarn Neelapaichit, Ronachai Kongsakon, Somporn Chotivitayataragorn, Umaporn Udomsubpayakul

**Affiliations:** 1 Ramathibodi School of Nursing Faculty of Medicine Ramathibodi Hospital, Faculty of Medicine Ramathibodi Hospital, Mahidol University, Bangkok, Thailand; 2 Department of Psychiatry, Faculty of Medicine Ramathibodi Hospital, Mahidol University, Bangkok, Thailand; 3 Department of Nursing, Faculty of Medicine Ramathibodi Hospital, Mahidol University, Bangkok, Thailand; 4 Department of Clinical Epidemiology and Biostatistics, Faculty of Medicine Ramathibodi Hospital, Mahidol University, Bangkok, Thailand

**Correction: BMC Women’s Health 23, 294 (2023)**

**https://doi.org/10.1186/s12905-023–02440-x**

In the sentence beginning ‘The survey was conducted............ December, 2020)’ in the Quantitative component heading under the Methods section of this article [1], was incorrect and should have read as ‘The researchers conducted the survey using questionnaires to assess family violence during two phases of the COVID-19 pandemic: at baseline (January to May 2020) and at six-month follow-up (July to December 2020).’

The original article has been corrected.
