# Supplementary material for: Impacts of COVID-19 on family violence in Thailand: prevalence and influencing factors
Source: BMC Womens Health. 2023 Jun 1;23:294. doi: 10.1186/s12905-023-02440-x (PMC10233183; doi:10.1186/s12905-023-02440-x)
Supplement: Supplementary file 1 — Additional file 1. [file 12905_2023_2440_MOESM1_ESM.docx]

**Semi-structure questions for a focus group interview**

1. What are the impacts of COVID-19 on your clients with family violence and their families?
2. What do you think of the frequency of family violence among your clients? Is it increase or decrease the number of family violence?
3. What are the factors that influence family violence during the COVID-19 pandemic?
4. What are the service limitations for victims (women and children living in the family) of family violence? How do you manage to help clients accessing to services during the COVID-19 pandemic?
