# Supplementary material for: Impacts of COVID-19 on family violence in Thailand: prevalence and influencing factors
Source: BMC Womens Health. 2023 Jun 1;23:294. doi: 10.1186/s12905-023-02440-x (PMC10233183; doi:10.1186/s12905-023-02440-x)
Supplement: Supplementary file 2 — Additional file 2. [file 12905_2023_2440_MOESM2_ESM.docx]

Family health assessment form in the COVID-19 outbreak

Please read or listen to the consent carefully and understand all information before you sign.

Do you agree to participate in this project?

 1. Yes ≤ 2.No

SECTION 1: GENERAL INFORMATION

1. Age………………years

2. Marital status

 1. Single

 2. Married/married together

 3. Married/married, separated (because of work)

 4. Married/married, separated (because of a bad relationship)

 5. Widow/divorce/breakup

3. Education level

 1. Under elementary school/not studying

 2. Elementary school

 3. Junior High School

 4. Higher Secondary School/Equivalent (Vocational Certificate)

 5. Diploma/Equivalent (High Vocational Certificate)

 6. Bachelor's degree or equivalent

 7. Master's degree or higher

4. Occupation

 1. Lay, persons,  2. Personal business/Shop

 3. Agriculture/fishery ≤ 4. Government officials/state enterprises/pensions

 5. Employee ≤ 6. Student/Student

 7. Housekeeper ≤ 8. Unemployed

 9. Others, specify ................................................ ..............

5. Average income per household (before the COVID-19 pandemic)

 1. Less than 5,000 baht ≤ 2. 5,001– 10,000 baht

 3. 10,001 – 20,000 baht ≤ 4. 20,001 – 30,000 baht

 5. 30,001 – 40,000 baht ≤ 6. 40,001 –50,000 baht

 7. Above 50,000 baht ≤ 8. Unknown

6. Average income per household (Current)

 1. Less than 5,000 baht ≤ 2. 5,001– 10,000 baht

 3. 10,001 – 20,000 baht ≤ 4. 20,001 – 30,000 baht

 5. 30,001 – 40,000 baht ≤ 6. 40,001 –50,000 baht

Before the COVID-19 pandemic, what do you think about your family income?

 1. Have more income than expenses

 2. Have enough income but have no savings

 3. Not having enough income for some months

 4. Not having enough income / no income

8. What do you think about your family's income each month?

 1. have more income than expenses

 2. Have enough income but have no savings

 3. Not having enough income for some months

 4. Not having enough income / no income

9. What kind of family type?

 1. Single Family

 2. extended family

 3. Single-parent family

 4. Middle cleft family

 5. Other (specify)................................................ ...............

10 Do you have children living with you in your family?

 1. Yes ≤ 2. No

11. Is there anyone in your family using drugs, smoking, or substance?

10.1 Smoking  1. Yes,  2. No

10.2 Drinking alcohol  1. Yes,  2. No

10.3 Any substances ≤ 1. Yes (please specify).................................................

2. none

12. Has anyone been infected with COVID-19 or has been in contact with an infected person?

 1. Yes ≤ 2. No

Part 2: Impact and Management from the COVID-19 Pandemic Situation

13. After the COVID-19 pandemic, someone in your family has been affected by work.

 1. Not affected

 2. Decrease in income

 3. Suspended or having to suspend business temporarily

 4. Being fired from work/laid off work/must stop doing business permanently

 5. Other (specify)................................................ ...............

14. After the COVID-19 pandemic, your family is affected economically.

 1. Not affected

 2. Low impact

 3. Moderate impact

 4. Huge impact

 5. Severe impact

15. After the COVID-19 pandemic, what is your solution if your family's economy is terrible?

 1. Don't have/don't know

 2. Yes, specify guidelines................................................ ..................................................

16. After the COVID-19 pandemic, has anyone in your household been affected by mental health, stress, or discomfort?

 1. Not affected

 2. Slightly affected

 3. Moderately affected

 4. Severely affected

 5. Don't know

17. Due to the COVID-19 pandemic, how is your family stressed?

 1. Stress as usual

 2. Slightly more stressful

 3. Quite a lot more stressed than before

 4. Very stressful

18. What is the level of your stress today?

**
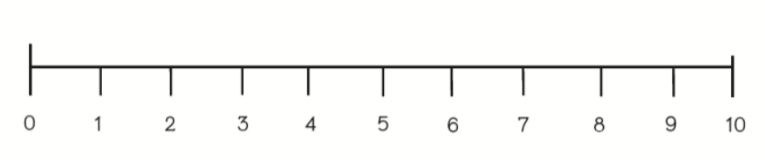
**

I am not stressed at all. Very stressful.

19. What is your family's way of reducing stress?

 1. I don't have/ don't know how to do it.

 2. Yes, specify guidelines................................................ ..................................................

Does your family have discussions and counseling about family problems?

1. none

 2. There is discussion, and there is a solution.

 3. Have a conversation, but there were conflicts in the family.

 4. Others, specify ................................................ .................................................. ......

21. Have you or someone in your family ever had suicidal thoughts?

 1. never

yes, I have

22. What do you think? with the children in the home not going to school for a long time

 1. There are no children in the house.

 2. It's good because reducing exposure to COVID-19.

 3. It is a burden because it increases the cost problem. or needs to be nurtured

 4. Others, specify ................................................ .................................................. ......

23. How long did you and your family adapt to the pandemic or city lockdown under the pandemic?

 1. Less than one month

 2. 1 month

 3. 2 months

 4. 3 months

 5. More than three months

24. How much longer do you think you and your family can return to everyday life?

 1. 1-3 months

 2. 3-6 months

 3. 6 months - 1 year

 4. At least one year

Part 3: The problem of domestic violence

25. Have you or a family member ever been emotionally abused by family members, such as insults, insults, or unfaithful behavior during the past three months?

 1. Yes, I have

 2. Never (skip to question 26)

25.1 If yes, Has your family been abused in the following ways? (more than one answer possible)

 1. Insult, criticize, humiliate

 2. Intimidation in various ways until it is unacceptable.

 3. Ignoring, cold, uninterested, causing regret.

 4. Show tantrums, destroy things, and hurt pets.

 5. Breaking a promise and not keeping your word cause a loss of trust

 6. having unfaithful behavior

 7. Domination/control

 8. Others (specify)………………………………

26. Have your family ever been physically abused by a family member in the last three months?

 1. Yes, I have

 2. Never (skip to question 27)

26.1 If yes, how much have you (or the victim) been injured?

 1. No injuries

 2. Injury but not severe

 3. Severe injuries – see a doctor

 4. Severe injury – not going to the doctor

26.2 If you or a member of The family had been beaten and beaten. Does the abuser use the method of Hurting in any of the following ways? (more than one answer)

 1. Use force to push, swing, pull, drag, or throw things at them.

 2. Use force to slap, punch, kick, stomp, or strangle.

 3. Assault with weapons such as sticks, knives, and guns.

 4. Make the body blister or burn from the use of cigarettes or use it to pour hot

 5. Use other methods (specify)………………………………………………

27. In the last three months, have you or a family member been subjected to sexual violence by members of your family (including your husband), such as sexual harassment, harassment, indecent, or rape?

 1. Yes, I have

 2. Never (end of interview)

27.1 If yes, you or your family member Have you ever been sexually violent by someone in your family (including your husband) in any of the following ways? (more than one answer)

 1. Speech, slander, sexually

 2. lewd/obscene

 3. Rape

 4. Other (specify)................................................
